# Novel digital technique for measuring the volumetric healing process of free gingival grafts surrounding dental implants

**DOI:** 10.3389/fdmed.2024.1372312

**Published:** 2024-07-02

**Authors:** Cristian Docampo-Vázquez, Teresa Gragera-Alia, Manuel Fernández-Domínguez, Álvaro Zubizarreta-Macho, Juan Manuel Aragoneses-Lamas

**Affiliations:** ^1^ Faculty of Dentistry, Alfonso X El Sabio University, Madrid, Spain; ^2^ Department of Surgery, Faculty of Medicine and Dentistry, University of Salamanca, Salamanca, Spain; ^3^ Department of Dentistry, Universidad Federico Henríquez y Carvajal, Santo Domingo, Dominican Republic

**Keywords:** dental implants, periodontics, morphometry, digital, free gingival graft, healing

## Abstract

The objective of the present study was to analyze and describe a new digital technique for analyzing the volumetric healing process of free gingival grafts in both donor and recipient locations surrounding a dental implant, as well as to compare the reliability of conventional and digital techniques for measuring the width of the free gingival graft in the recipient location throughout the healing process.

**Materials and methods:**

Ten patients presenting with mucositis linked to a dental implant were included. A preoperative soft tissue width <2 mm, with probing pocket depth <5 mm, edema and inflammation and bleeding on probing was determined A digital impression was taken of both donor and recipient locations using an intraoral scan, generating a Standard Tessellation Language digital file both preoperatively (STL1) and after 1 week (STL2), 1 month (STL3), 3 months (STL4), and 6 months (STL5) of follow-up. Afterwards, the digital files (STL1-STL5) were aligned using a reverse engineering morphometric software, and Student's *t-*test was used to analyze changes in volume at the donor and recipient locations. Additionally, widths were measured both clinically and digitally so as to compare the reliability of these measurement techniques. The repeatability and reproducibility of both these measurement techniques were also analyzed using Gage R&R statistical analysis.

**Results:**

Gage R&R found that the total variability of the digital technique was 0.6% (among the measures of each operator) and 7.6% (among operators); as variability was under 10%, the results were repeatable and reproducible. In addition, there were statistically significant differences between donor and recipient locations in healing process volume (mm^3^) after one week (*p* = 0.0110), one month (*p* = 0.0007), three months (*p* < 0.0001) and six months (*p* = 0.0004) of follow-up.

**Conclusion:**

The digital measurement technique provided accurate, repeatable, and reproducible results when analyzing the volumetric and linear measures of the healing process in both the donor and recipient locations of a free gingival graft surrounding a dental implant, with significantly higher tissue volume in the recipient location.

## Background

Dental implants are currently considered a highly predictable treatment option for the rehabilitation of partial or total edentulism, with a reported survival rate of 97% (1). Furthermore, Klinge et al. reported increasing rates of dental implant placement in the world, with between 12 and 18 million dental implants placed annually (2). However, bacterial infection causes plural inflammation around dental implants, which can result in the appearance of mucositis and therefore peri-implant affection that negatively impacts dental implant survival (3, 4).

Unfortunately, peri-implant diseases are increasing in patients, with figures around 22% (5); therefore, effectively managing peri-implant mucositis is an important measure for clinicians to take in order to prevent peri-implantitis (6). Giannobile et al. reported that maintaining sufficient thickness and width of keratinized tissue surrounding dental implants appears to be an important factor in the prevention of peri-implant diseases (7), although this width around dental implants involves a complex biological process that requires weeks of healing to form. Furthermore, the width provides a biological barrier against bacteria, allowing both soft and hard tissues to be remodeled around dental implants (8). Indeed, Perussolo et al. highlighted the effect of 2 mm of keratinized tissue surrounding dental implants in maintaining peri-implant tissue health, compared with dental implants surrounded by less than 2 mm of keratinized tissue, which were more susceptible to biological complications in the peri-implant tissues (9). Furthermore, Thoma et al. reported that autogenous grafts used for soft tissue augmentation provide the most predictable maintenance of peri-implant tissue health, as they increase the width and thickness of keratinized tissue in dental implants (10).

Additional techniques may help improve the quality and width of the soft tissue surrounding dental implants, including free gingival grafts (FGGs), which increase keratinized mucosa, connective tissue grafts (CTGs), which in turn improve aesthetic results (11), and autogenous graft substitutes (12), which are frequently used to minimize postoperative mucosal recession and improve tissue thickness during immediate placement of dental implants (13). Additionally, FGGs have been also indicated to re-establish an adequate keratinized tissue width and gingival thickness in the presence of mucogingival defects (14, 15) both in natural teeth and dental implants (7, 16). The FGG technique is considered the best treatment option for augmenting the thickness of soft tissue and keratinized tissue/mucosa in teeth and at sites of dental implants (15). Roccuzzo et al. reported that the use of FGG in procedures for augmenting soft tissue resulted in reduced mucosal inflammation, improved patient comfort, and enabled better control of plaque around dental implants without keratinized tissue (17); however, visual measurement procedures or those using a periodontal probe have been used to analyze the degree of success of gingival grafts or the healing process at both the donor and recipient sites. A single study examined the volumetric changes, concluding that the increase in soft tissue using grafts with a collagen matrix is similar to that achieved with autogenous subepithelial connective tissue grafts, but this was measured in beagle dogs (18).

Previously, Marques et al. conducted a pilot study to evaluate digitally the healing dynamics process of the hard palate after free gingival graft (19); moreover, Ramos et al. performed a prospective cohort study to compare the healing pattern in the lateral palate following harvesting of connective tissue graft at 3 and 6 months postoperatively by two different harvesting techniques (20), and Tavelli et al. reported the volumetric changes that occur at the palatal donor site after harvesting a soft tissue graft using a digital measurement procedure (21).

The objective of the present pilot study was to analyze and describe a new digital technique for analyzing the volumetric healing process of free gingival grafts in both donor and recipient locations surrounding a dental implant, as well as to compare the reliability of conventional and digital techniques for measuring the width of the free gingival graft in the recipient location throughout the healing process, with a null hypothesis (H_0_) stating that the free gingival graft does not change the volume in either donor or recipient locations throughout the healing process, and digital and conventional techniques provide similar reliability when measuring the width of the free gingival graft in the recipient location throughout the healing process.

## Methods

### Study design

A pilot clinical trial was conducted between January and September 2022 at the Dental Centre of Innovation and Advanced Specialties at Alfonso X El Sabio University, in compliance with the ethical standards of the Declaration of Helsinki and the CONSORT Statement. The Ethical Committee of the Faculty of Health Sciences at University Alfonso X El Sabio authorized the study in December 2021 (process no. 29/2021). Informed consent was granted by all patients prior to provision of their digital files. The repeatability and reproducibility of this novel technique for measuring the volumetric healing process of free gingival grafts around dental implants have been analyzed using Gage R&R statistical analysis. This technique has been previously used by the authors to analyze the wear of screw-retained implant-supported metal-ceramic dental prostheses and natural tooth as antagonist (22), the distal tooth displacement and derotation angle produced by the Carriere Motion Appliance (23), the wear of the bracket slot walls of the fixed multibracket appliance after orthodontic treatment (24), the volume of maxillary and nasal sinus airways following suture palatine expansion performed with the Hyrax disyuntor appliance (25) and the wear volume of controlled memory (CM)-wire NiTi alloy endodontic reciprocating files after clinical use (26), the volumes of the left and right maxillary sinuses and the nasal and maxillary sinus airway complex after a sinus lift procedure using the lateral window approach (27), the volume of the midpalatal suture after rapid maxillary expansion (28) and the area and volume of the remaining cement after removal of fixed multibracket appliances, the area and volume of remaining cement after cement removal, the area and volume of enamel removed after cement removal, and the volume of cement used to adhere fixed multibracket appliances (29), with a sample size of two operators and two repetitions per operator, since Carrion García et al. (30) and Zanobini et al. (31) suggested that this sample size is sufficient to demonstrate its usefulness for evaluating the precision and consistency of a measurement process. Specifically, the Gage R&R determines how much of the variability in the measurement process is due to variation in the measurement system; You use inference techniques to estimate repeatability and reproducibility. When a measurement process is conducted, the total process variation consists of part-to-part variation plus measurement system variation. Measurement system variation is determined by the repeatability, which is described as the variability of the measures performed by the same operator when the same part is measured, and the reproducibility, which is the variability of the measures performed by different operators when the same part is measured. Ideally, very little of the variability should be due to repeatability and reproducibility. Differences between parts (part-to-part) should account for most of the variability. When variability occurs, the measurement system can reliably distinguish between parts.

### Clinical procedure

Ten patients presenting with mucositis associated with a dental implant located in the lower maxilla to support an overdenture were referred to the Master's Degree of Oral Implantology and Implant-Supported Prostheses at Alfonso X El Sabio University (Madrid, Spain). Inclusion criteria were determined to select patients with preoperative soft tissue width <2 mm, probing pocket depth <5 mm, edema and inflammation and bleeding on probing (32). The bacterial load present around the dental implants was eliminated and patients were recommended to use an oral mouth rinse-based chlorhexidine digluconate twice a day, and follow-up appointments were scheduled. Subsequently, a free gingival graft was planned to increase the volume surrounding the dental implant, and a 3 × 2-cm free gingival graft was extracted from the palatal region before being placed in the buccal gingival surface of the surrounding dental implants. The patients were scheduled for follow-up appointments at 1 week (STL2), 1 month (STL3), 3 months (STL4), and 6 months (STL5).

### Experimental procedure

**Figure 1:**
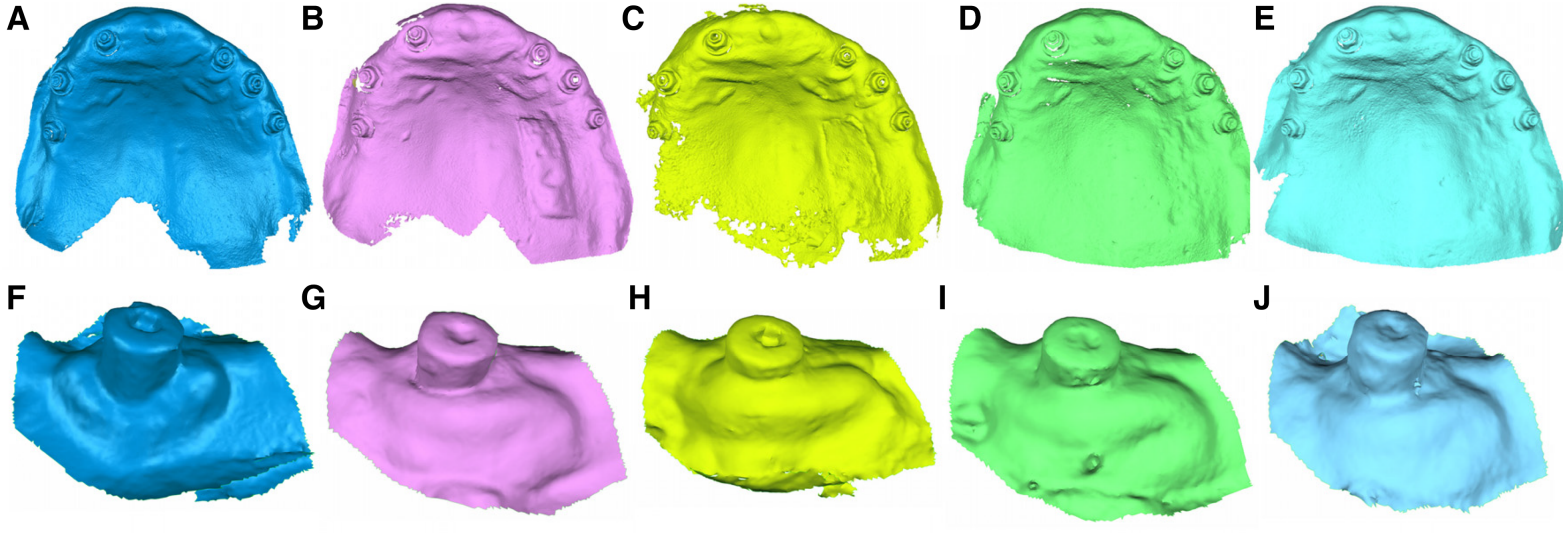
(**A**) Preoperative, (**B**) 1-week follow-up, (**C**) 1-month follow-up, (**D**) 3-month follow-up, and (**E**) 6-month follow-up STL digital files of the donor region of the free gingival graft from the palate. (**F**) Preoperative, (**G**) 1-week follow-up, (**H**) 1-month follow-up, (**I**) 3-month follow-up, and (**J**) 6-month follow-up STL digital files of the recipient location.

A preoperative digital impression was taken of both donor and recipient locations using 3D in-motion video imaging technology (Figures 1A,F) through an intraoral scan (True Definition, 3M ESPE ™, Saint Paul, MN, USA) to create a standard tessellation language (STL) digital file (STL1) with a cloud of points forming a tessella network, with 3-dimensional objects represented as polygons comprising tessellas in the form of equilateral triangles (33, 34). The image-capturing procedure was carried out in accordance with the manufacturer's recommendations by first scanning the area of interest and then the rest of the dental arch surface. The patients were scheduled for follow-up appointments at 1 week (STL2) (Figures 1B,G), 1 month (STL3) (Figures 1C,H), 3 months (STL4) (Figures 1D,I) and 6 months (STL5) (Figures 1E,J), during which postoperative digital impressions were taken using an intraoral scan (True Definition, 3M ESPE ™, Saint Paul, MN, USA).

### Alignment procedure

**Figure 2:**
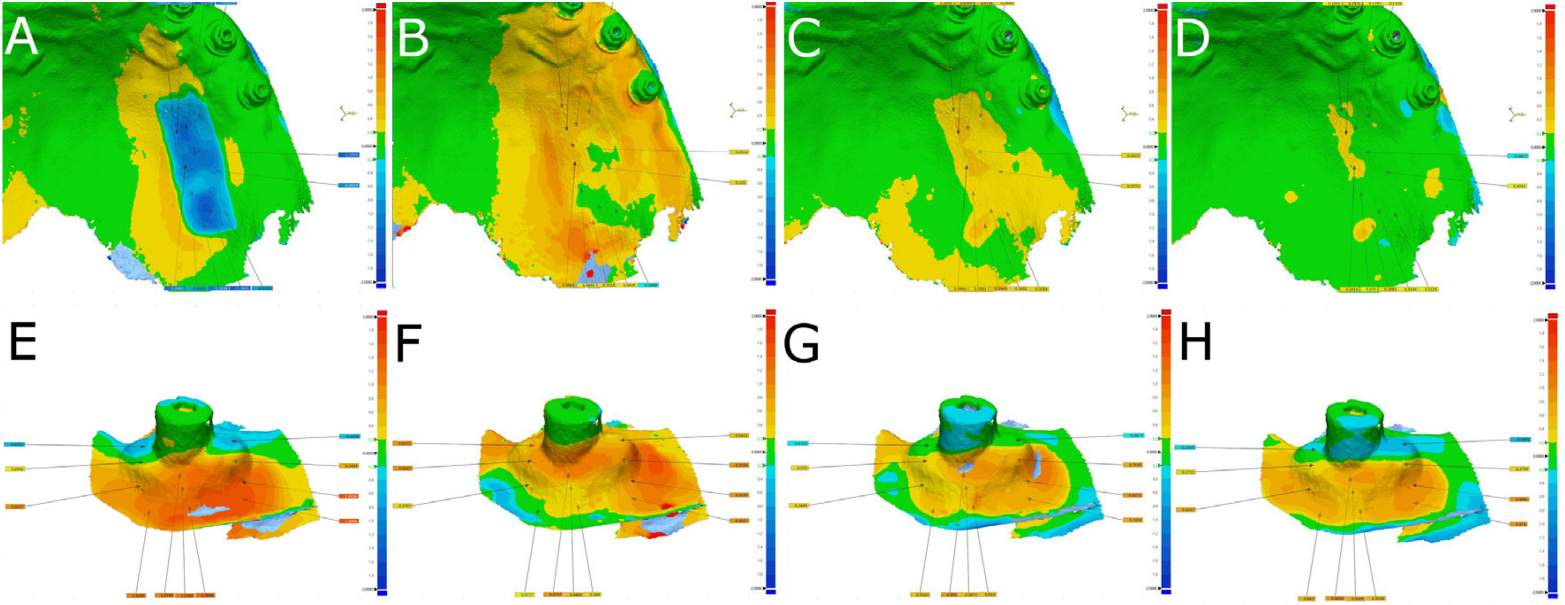
(**A**) Alignment and measurement procedure between STL1 and STL2, (**B**) STL1 and STL3, (**C**) STL1 and STL4, and (**D**) STL1 and STL5 digital files of the donor region of the free gingival graft from the palate. (**E**) Alignment and measurement procedure between STL1 and STL2, (**F**) STL1 and STL3, (**G**) STL1 and STL4, and (**H**) STL1 and STL5 digital files of the recipient location. Warm colors indicate an increase in volume, cold colors indicate a decrease in volume, and green indicates an accurate alignment.

After importing STL1–5 of both donor and recipient locations to a reverse engineering morphometric software (3D Geomagic Capture Wrap, 3D Systems©, Rock Hill, SC, USA), which was used to conduct a full-arch alignment procedure. STL1 of both donor and recipient locations were used as a reference, with STL2–5 overlaid on top using the best fit algorithm. Next, the STL1 digital file was segmented and separately compared in 3D with the STL2 digital file (Figures 2A,E), STL3 digital file (Figures 2B,F), STL4 digital file (Figures 2C,G), and STL5 digital file (Figures 2D,H); tolerance was set at ±10 µm and spectrum at ±100 µm.

### Digital measurement procedure

**Figure 3:**
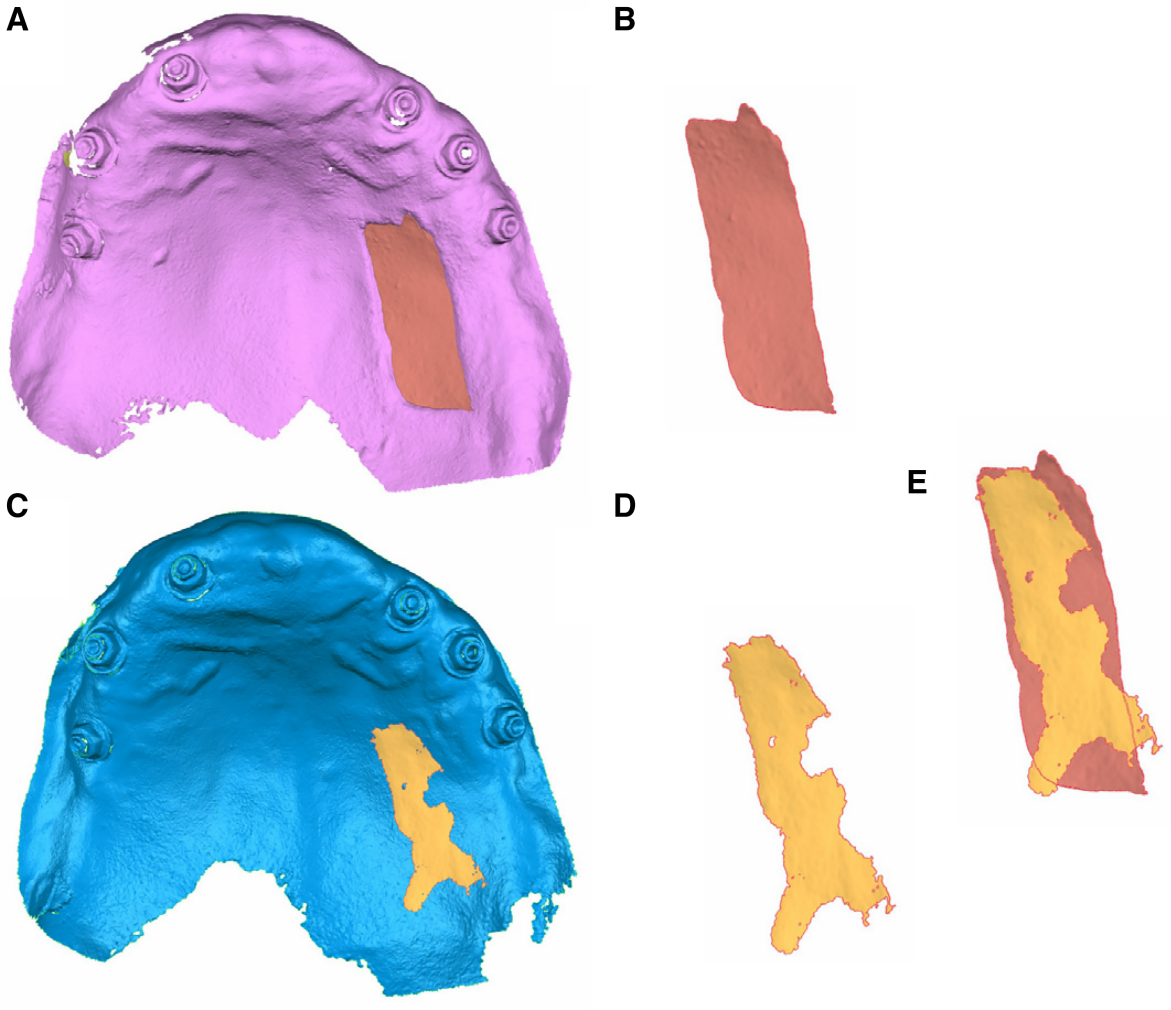
(**A**) Palatal view of the STL1 and STL2 digital files of the donor location and (**B**) isolated volume of the free gingival graft surrounding the dental implant. (**C**) Palatal view of the STL1 and STL5 digital files of the donor location and (**D**) isolated volume of tissue in the process of healing. (**E**) Comparative analysis of the isolated volumes.

After aligning the files, volume changes at the donor and recipient locations after the free gingival graft surrounding the dental implant were measured at the 1-week (STL2), 1-month (STL3), 3-month (STL4), and 6-month (STL5) follow-up appointments. In addition, changes in volume after the free gingival graft surrounding a dental implant at the donor and recipient locations were isolated to enable accurate measurement throughout the 6-month healing process (Figure 3).

**Figure 4:**
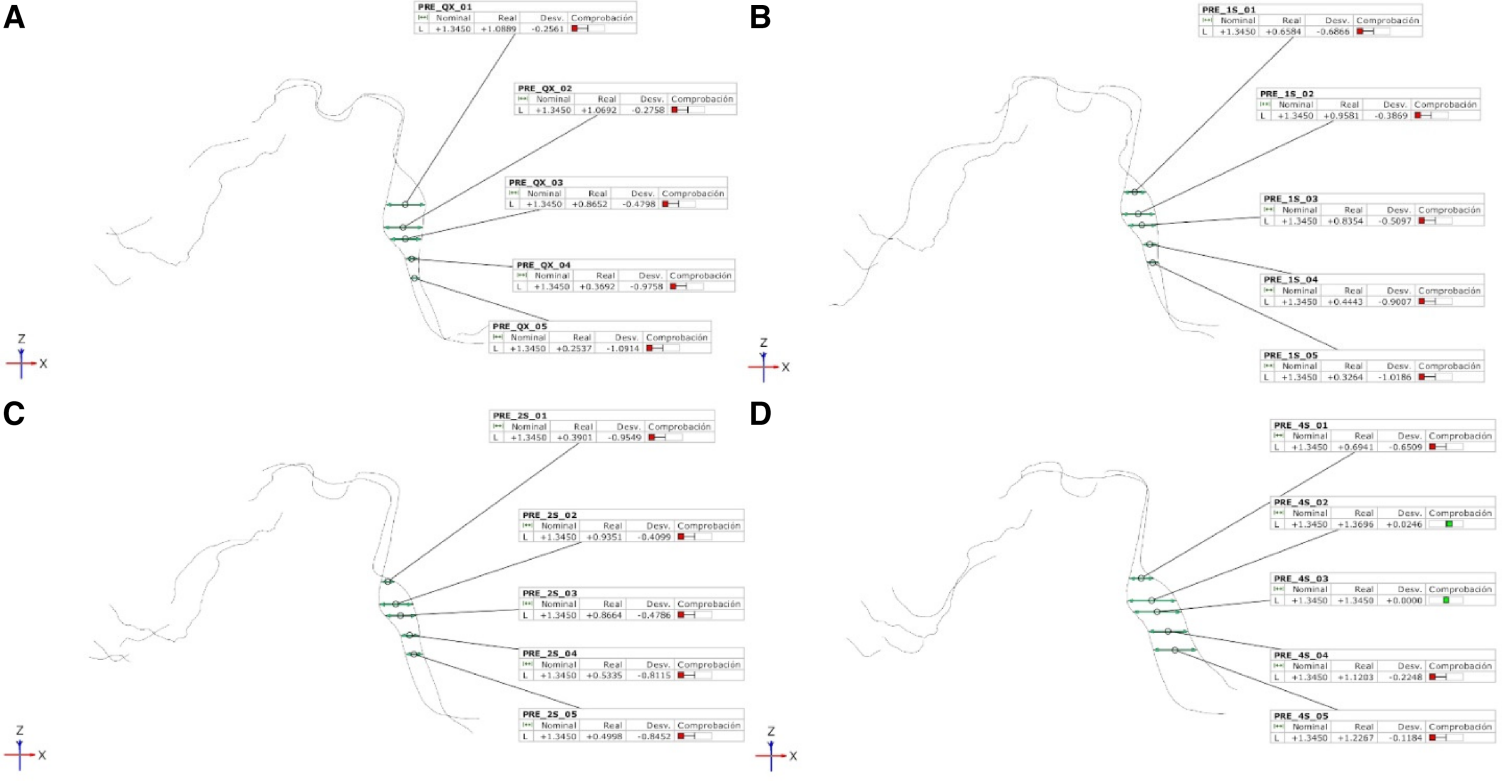
(**A**) Mesial view of the alignment procedure between STL1 and STL2, (**B**) STL1 and STL3, (**C**) STL1 and STL4, and (**D**) STL1 and STL5 digital files to measure the width of the healing process of the free gingival graft surrounding the dental implant.

In addition, linear measurements (mm) of the width of the healing process of the free gingival graft surrounding a dental implant were also taken in the recipient locations at 1 week (Figure 4A), 1 month (Figure 4B), 3 months (Figure 4C) and 6 months (Figure 4D) of follow-up after aligning the digital files with the preoperative STL digital file using engineering morphometric software (3D Geomagic Capture Wrap, 3D Systems©, Rock Hill, SC, USA).

### Clinical measurement procedure

In addition, the thickness of the healing process of the free gingival graft surrounding a dental implant was clinically measured following the procedure used by Huang et al. (35), by using an endodontic file with a rubber stop to measure the thickness of the free gingival graft at the mid-buccal aspect in the middle point of the apical–coronal direction.

### Confirming repeatability and reproducibility

To confirm the repeatability of both the new and conventional measurement techniques, the same operator (Operator A) calculated the aforementioned measurements. Another operator (Operator B) calculated the measurements twice to confirm the reproducibility of both the new and the conventional measurement techniques.

### Statistical tests

SAS 9.4 (SAS Institute Inc., Cary, NC, USA) was used to carry out statistical analysis of the measurement variables. Descriptive statistics were expressed as mean and SD for quantitative variables. Given the standard distribution of the variables, Student's *t-*test was used to compare the volume (mm^3^) of the healing process of a free gingival graft in both donor and recipient locations surrounding a dental implant for comparative analysis. Comparative analysis was also performed by comparing digital and conventional measurement techniques with regard to the linear measurement (mm) of the free gingival graft width in recipient locations surrounding a dental implant. *P* < 0.05 was used to determine statistical significance. Analysis of the repeatability and reproducibility of both the new and conventional measurement techniques was conducted using Gage R&R statistical analysis.

## Results

**Figure 5:**
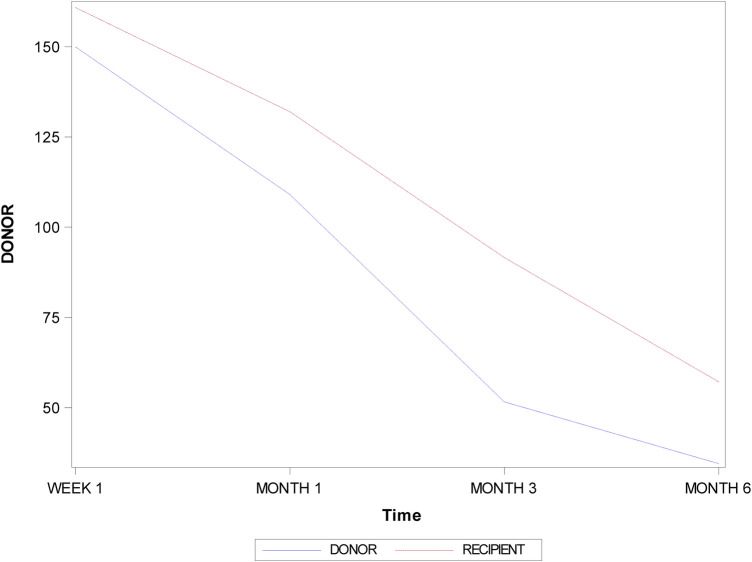
Evolution of the volume (mm^3^) of the healing process between donor and recipient locations after one week, one month, three months, and six months.

Table 1 and Figure 5 display the mean and SD values for the volume (mm^3^) of the healing process in both donor and recipient locations of a free gingival graft surrounding a dental implant.

**Table 1:** Descriptive statistics of the volume (mm^3^) of the healing process of a free gingival graft in both donor and recipient locations surrounding a dental implant.

Moment	Location	*n*	Mean	SD	Minimum	Maximum
1 Week	Donor	10	149.94	7.88	135.00	160.20
	Recipient	10	160.82	7.43	147.30	170.60
1 Month	Donor	10	109.05	7.61	98.10	119.30
	Recipient	10	131.97	8.32	116.20	142.70
3 Months	Donor	10	51.58	7.01	38.30	62.40
	Recipient	10	91.58	5.50	82.90	99.00
6 Months	Donor	10	34.48	8.25	24.20	52.90
	Recipient	10	57.15	7.79	47.10	67.60

There were statistically significant differences in the volume (mm^3^) of the healing process between donor and recipient locations after one week (*p* = 0.0110), one month (*p* = 0.0007), three months (*p* < 0.0001), and six months (*p* = 0.0004) (Figure 5).

**Figure 6:**
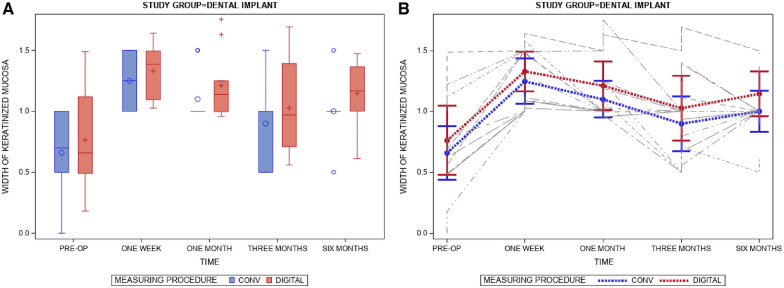
(**A**) Box plot of the digital and conventional linear measurement (mm) techniques during the follow-up appointments. The respective median value of the study groups is represented by a horizontal line in each box. +,◦; Mean value of the box plots. (**B**) Error bar plot of the digital and conventional linear measurement (mm) techniques during the follow-up appointments. Grey lines represent each individual measurement.

Table 2 and Figure 6 compare the mean and SD values between digital and conventional measurement techniques for the linear measurements (mm) of the width of the free gingival graft in recipient locations surrounding a dental implant throughout the healing process.

**Table 2:** Descriptive statistics of the linear measurement (mm) of the width of the free gingival graft during the healing process in recipient locations surrounding a dental implant compared between digital and conventional measurement techniques.

Measure	Time	*n*	Mean	SD	Minimum	Maximum
Conventional	Pre-op	10	0.66	0.31	0.00	1.00
	One week	10	1.25	0.26	1.00	1.50
	One month	10	1.10	0.21	1.00	1.50
	Three months	10	0.90	0.32	0.50	1.50
	Six months	10	1.00	0.24	0.50	1.50
Digital	Pre-op	10	0.76	0.40	0.18	1.49
	One week	10	1.33	0.23	1.03	1.64
	One month	10	1.21	0.28	0.96	1.75
	Three months	10	1.03	0.37	0.56	1.69
	Six months	10	1.14	0.26	0.61	1.47

There were no statistically significant differences between digital and conventional measurement techniques in the linear measurement (mm) of the width of the free gingival graft in recipient locations surrounding a dental implant throughout the healing process at the preoperative assessment (*p* = 0.3252) and one-week (*p* = 0.4525), one-month (*p* = 0.2915), three-month (*p* = 2,268), and six-month (*p* = 0.1705) follow-up (Figure 6A,B).

**Figure 7:**
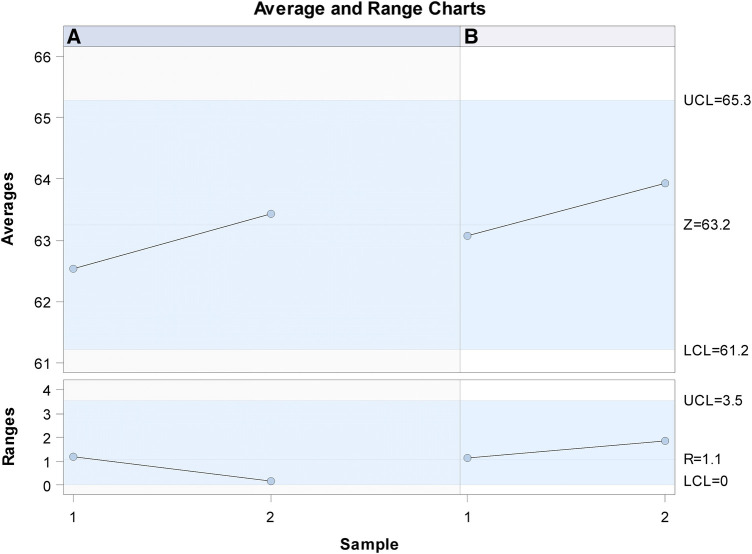
Charts of the average of the two volume (mm^3^) measurements of the healing process of a free gingival graft surrounding a dental implant.

**Figure 8:**
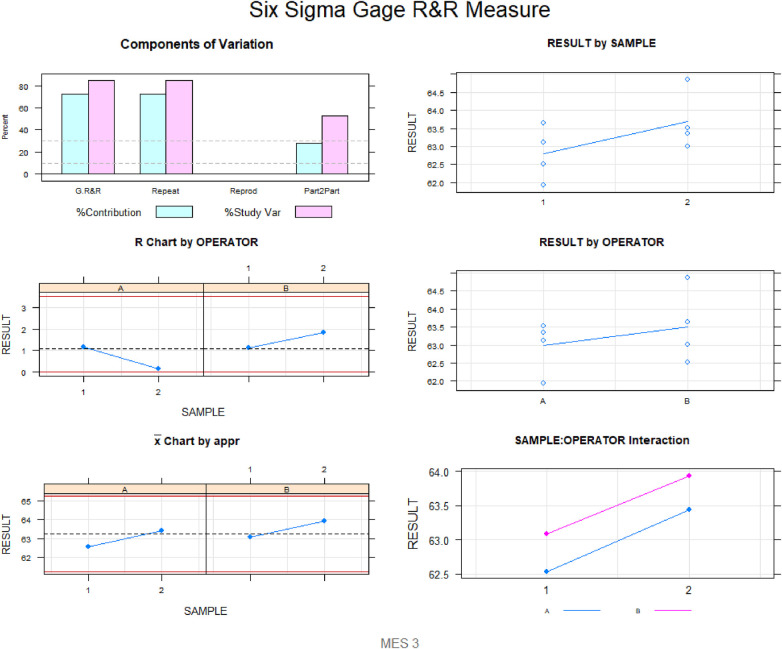
Measurement system analysis of the volume (mm^3^) of the healing process of a free gingival graft surrounding a dental implant with a chart displaying how each component contributes to the total variance (components of variation); a mean control chart, and a range control chart (R chart by operator and x chart by appr); every measurement point in the graph (trial by I and trial by operator II); and the interactions between the operators (i: operator interaction).

Gage R&R statistical analysis of the volume (mm^3^) of the healing process of a free gingival graft surrounding a dental implant measured using the digital measurement technique found that the variability attributable to the digital measurement technique was 0.6% (between the measurements of each operator) and 7.6% (between the measurements of both operators) of the total variability of the samples. As the variability was under 10% in both cases, the digital measurement technique for measuring the volume (mm^3^) of the healing process of a free gingival graft surrounding a dental implant was considered repeatable and reproducible (Figures 7, 8).

**Figure 9:**
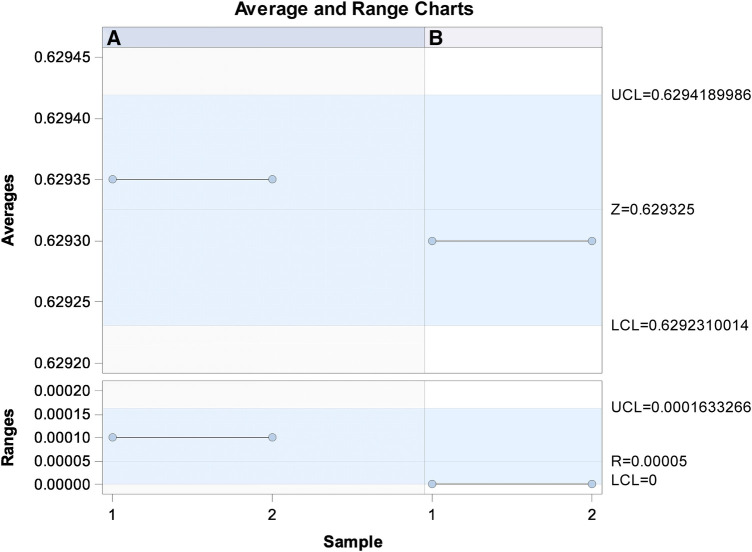
Charts of the average of the two width (mm) measurements of the healing process of a free gingival graft surrounding a dental implant, using the digital measurement technique.

**Figure 10:**
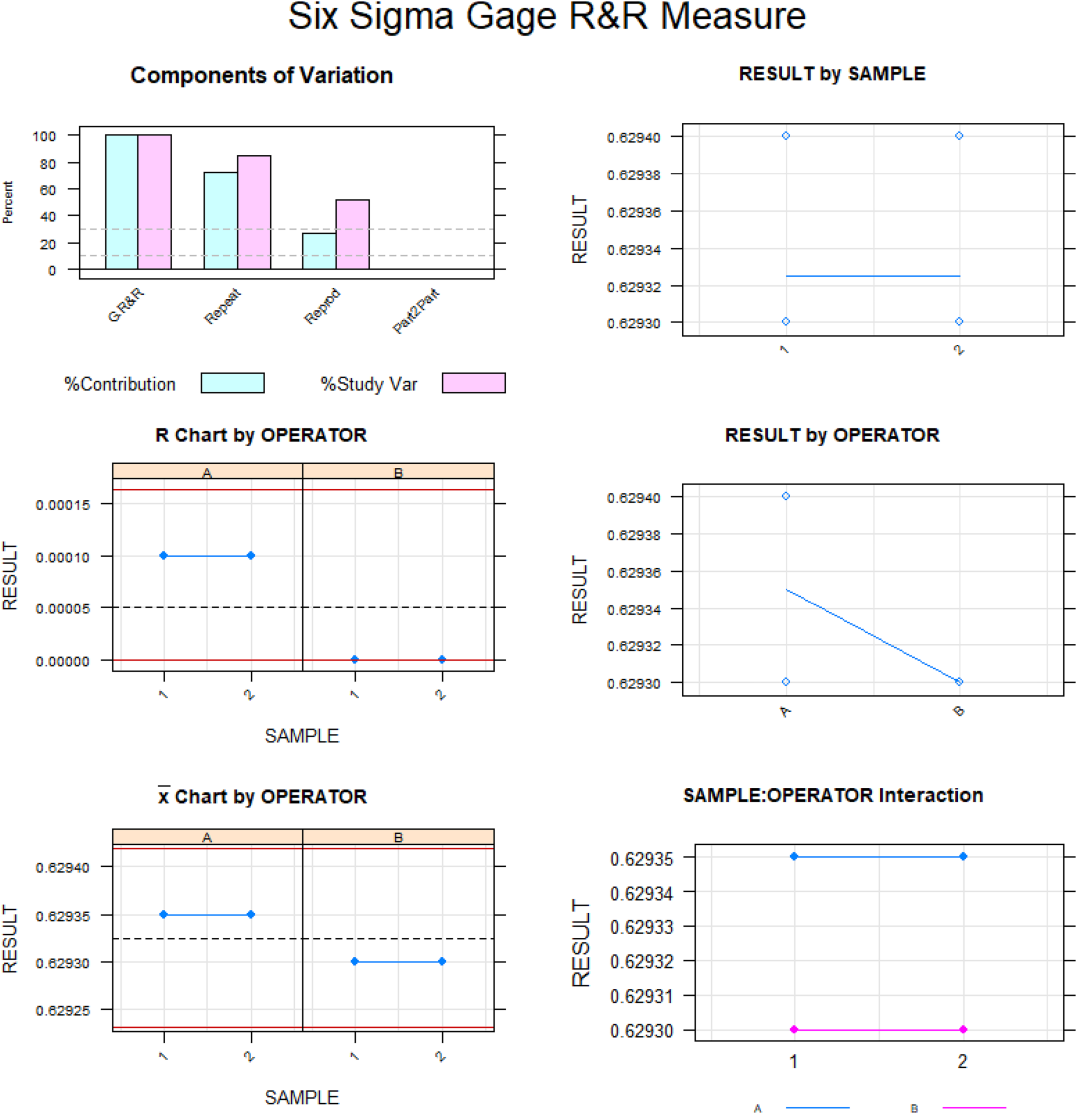
Measurement system analysis of the width (mm) of the healing process of a free gingival graft surrounding a dental implant, using the digital measurement technique, with a chart of how each component contributes to the total variance (components of variation); a mean control chart and a range control chart (R chart by operator and x chart by appr); every measurement point in the graph (trial by I and trial by operator II); and the interactions between the operators (i: operator interaction).

Gage R&R statistical analysis of the width (mm) of the healing process of a free gingival graft surrounding a dental implant measured using the digital measurement technique found that the variability attributable to the digital measurement technique was 2.7% (between the measurements of each operator) and 7.3% (between the measurements of both operators) of the total variability of the samples. As the variability was under 10% in both cases, the digital measurement technique for measuring the width (mm) of the healing process of a free gingival graft surrounding a dental implant was considered repeatable and reproducible (Figures 9, 10).

**Figure 11:**
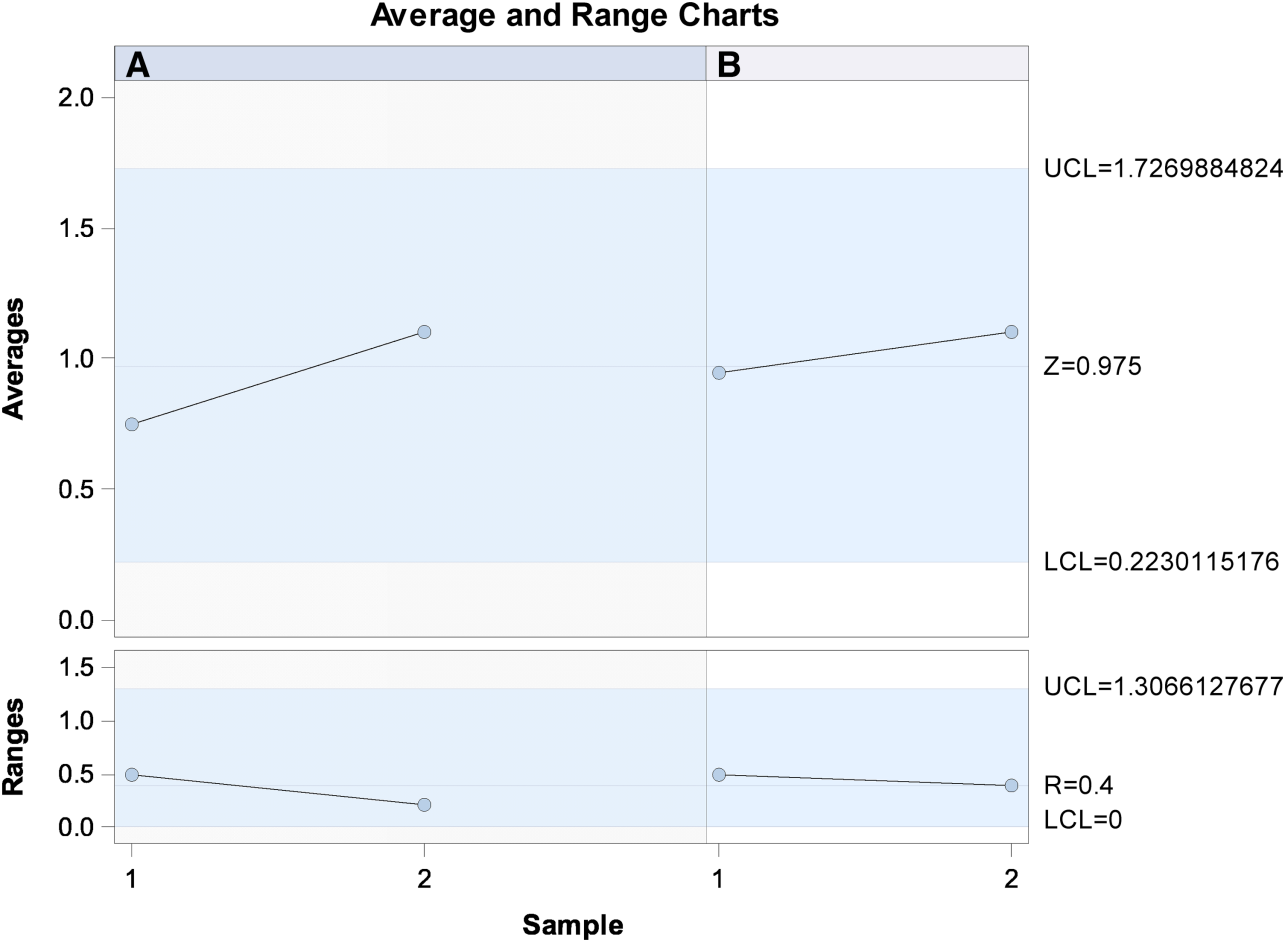
Charts for the average of the two width (mm) measurements of the healing process of a free gingival graft surrounding a dental implant, using the conventional measurement technique.

**Figure 12:**
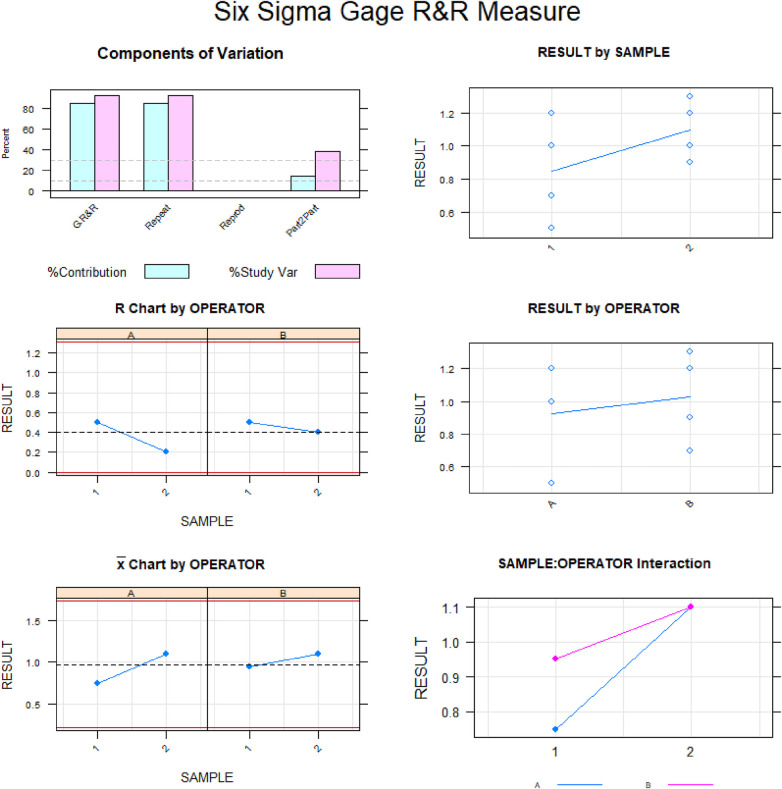
Measurement system analysis of the width (mm) of the healing process of a free gingival graft surrounding a dental implant, using the conventional measurement technique, with a chart of how each component contributes to the total variance (components of variation); a mean control chart and a range control chart (R chart by operator and x chart by appr); every measurement point in the graph (trial by I and trial by operator II); and the interactions between the operators (i: operator interaction).

Gage R&R statistical analysis of the width (mm) of the healing process of a free gingival graft surrounding a dental implant measured using the digital measurement technique found that the variability attributable to the conventional measurement technique was 85.3% (between the measurements of each operator) and 0.0% (between the measurements of both operators) of the total variability of the samples. As the variability was over 10%, the conventional measurement technique for measuring the width (mm) of the healing process of a free gingival graft surrounding a dental implant was considered reproducible but not repeatable (Figures 11, 12).

## Discussion

The results of this present study reject the null hypothesis (H_0_) that the free gingival graft does not change the volume in either donor or recipient locations throughout the healing process, and digital and conventional techniques provide similar reliability when measuring the width of the free gingival graft in the recipient location throughout the healing process.

This study indicates that the recipient location of free gingival grafts showed significantly higher tissue volume in comparison with the donor location. Additionally, the conventional linear measurement technique did not provide repeatable and reproducible measurements and should therefore not be recommended.

Mucogingival surgery procedures have been highly recommended to correct alterations affecting the position, morphology, and/or volume of the gingiva surrounding both teeth and implants (36); however, in clinical practice, connective tissue graft procedures, including harvesting and transplantation, require a thorough understanding of donor site anatomy and of the revascularization and tissue integration processes (37). Soft tissue grafting has become a widely used treatment option for increasing width and volume of keratinized soft tissue around dental implants, between 2.41 mm–3.1 mm (10), and it is associated with statistically significant differences (*p* < 0.001) in improved reduction of gingival and plaque index when compared with non-augmented sites. Initially, this surgical approach was used to augment the width of keratinized gingiva and the volume of the edentulous ridge (38); however, the high long-term outcomes (13.06 mm ± 2.26 mm after 5 years) (39) of this surgical approach extended its use to root coverage, augmentation of soft tissue surrounding dental implants, papilla reconstruction, partially edentulous areas (40), and scar correction (41). Furthermore, Thoma et al. compared the width of keratinized gingiva, morbidity, and surgery time between subepithelial connective tissue grafts, free gingival grafts, collagen matrix with an apically positioned flap/vestibuloplasty, and apically positioned flap and vestibuloplasty alone, finding statistically significant results indicating that the free gingival graft plus apically positioned flap and vestibuloplasty and the connective tissue graft were the most successful (*p* < 0.05) procedures for increasing keratinized mucosa width (qualitatively measured), and collagen matrix resulted in reduced surgery time and less morbidity; CTG surgery lasted 16 min longer than CMX, and time to recovery in terms of Oral Health Impact Profile (OHIP) scores was 6.0 ± 4.1 days in the CTG groups and 4.2 ± 3.4 days in the CMX group. This difference of 1.8 days was statistically significant (*p* < 0.05). Furthermore, free gingival grafts resulted in a statistically significant increase (*p* = 0.009) in the width of keratinized tissue (0.83 ± 0.61 mm) compared with the acellular dermal matrix. However, the acellular dermal matrix showed more tissue contraction (59.6%) whereas the free gingival graft presented a creeping attachment (17.6%) (15).

Additionally, autologous gingival grafts have shown high clinical outcomes and have therefore been highly recommended in terms of both gingival thickness and width of keratinized tissue. When comparing keratinized tissue with autogenous connective tissue in comparison with collagen matrices, the mean width gain was 0.62 mm higher (from 1.09 to 0.15 mm; CI 95%) (*p* < 0.001). The mean amount of gingival thickness gained was 0.32 mm (from 0.49 to 0.16 mm; CI 95%) higher after autogenous connective tissue grafts than after using collagen matrices (17). However, there were no statistically significant differences (*p* = 0.64) in donor areas of the autologous gingival grafts between the volume gain of the palate and the tuberosity gingival graft for root coverage (42), although pain levels were significantly lower in the tuberosity donor site than in the palatal donor site two weeks after the procedure (2.6 ± 2.16 vs. 5.9 ± 2.74, respectively; *p* < 0.001).

Accurate measurement of changes in volume after mucogingival surgery procedures and long-term stability of soft tissue autografts remain a concern (43). Previous studies have analyzed the volumetric outcomes of mucogingival surgery procedures using visual perception of the operator or millimeter probes (44, 45), but this measurement technique relies on the subjective perception of the operator and inaccurate elements, in addition to the inability to measure variations in volume; therefore, a novel non-invasive measurement technique for analyzing and comparing the healing progress of both donor and recipient sites in area and volume is proposed, which encourages its application in further studies comparing healing progress and volumetric changes after different mucogingival surgery procedures or gingival grafts.

To date, most articles analyze keratinized tissue width, soft tissue thickness, probing depth, recession depth, and clinical attachment level (42, 43, 45) using subjective linear measurements, whereas the proposed digital measurement procedure provides an objective, accurate, repeatable, and reproducible protocol for analyzing healing process of a free gingival graft surrounding a dental implant in both donor and recipient locations. However, this novel digital measurement technique requires using an intraoral scan to take a digital impression, and the resolution of this electronic device and accuracy of alignment could influence the measurement results; the digital measurement procedure established a spectrum of ±100 µm and tolerance of ±10 µm. Additionally, this digital procedure has been previously used to analyze DICOM–DICOM vs. DICOM–STL (46), two different protocols for matching data and creating surgical templates, in terms of clinical accuracy, analyzing the changes in volume that occur after placing implants in sites augmented with soft tissue compared vs. non-augmented sites (47). It has also been used to analyze the reliability of the representation of the alveolar process *in vivo* when using two intraoral surface scanners (48), although repeatability and reproducibility were not analyzed. In addition, while the accuracy and reliability of measurements of keratinized tissue taken using digital vs. conventional clinical techniques have been also analyzed, morphometric measurement techniques were not used (49).

The present study showed some limitations such as not including the volumetric healing process of free gingival grafts in both donor and recipient locations surrounding teeth, as well as not comparing other regenerative periodontal techniques or materials; however, the authors will analyze these concepts in further studies. On the other side, this study presents strength points since it provides an accurate, repeatable, reproducible and non-invasive digital measurement technique to assess the healing process of a free gingival graft surrounding a dental implant in both donor and recipient locations both linearly and volumetrically, which can be used for further studies.

## Conclusions

The present study indicates that the digital measurement technique provided accurate, repeatable, and reproducible results when analyzing the volumetric and linear measurements of the healing process of a free gingival graft surrounding a dental implant in both donor and recipient locations, with a significantly higher tissue volume in the recipient location.

## Data Availability

The raw data supporting the conclusions of this article will be made available by the authors, without undue reservation.
